# Cohort Profile: Growing Up in Wales: The Environments for Healthy Living study

**DOI:** 10.1093/ije/dyv178

**Published:** 2015-10-01

**Authors:** Kelly L Morgan, Ashrafunnesa Khanom, Rebecca A Hill, Ronan A Lyons, Sinead T Brophy

**Affiliations:** ^1^ Centre for the Development and Evaluation of Complex Interventions for Public Health Improvement (DECIPHer), School of Social Sciences, Cardiff University, Cardiff, UK,; ^2^ Farr Institute, College of Medicine, Swansea University, Swansea, UK and; ^3^ Public Health Wales NHS Trust, Cardiff, UK

## Why was the cohort set up?

Growing Up in Wales was established to examine the impact of gestational and postnatal environmental risk factors on infant health outcomes and to identify where structural change could be implemented to optimize health outcomes. In response to a call for the enhancement of existing longitudinal research ^1^ and with support from Public Health Wales, the study ‘Growing Up in Wales’ was set up. This study aimed to examine factors promoting health and to address known knowledge gaps and complement other epidemiology cohort studies around the world ^2–5^ by: (i) gathering prospective measurements early in the life course, therefore obtaining prenatal data rather than conducting baseline measurements after birth; and (ii) using objective measures, e.g. accelerometers, as opposed to relying on self-reported data for physical activity measurements. Initially the study set out to examine the impact of gestational and postnatal environments on offspring health and to examine where structural change can be brought about to optimize health outcomes. Adopting a multi-level analytical approach, the main objectives were to increase our understanding of interactions between: (i) intrauterine exposures, (ii) obesogenic environments and (iii) parental influences, on postnatal growth and development, diabetes, obesity and unintentional injuries.

Between November 2009 and March 2015, women were recruited from antenatal clinics based within the hospital and general practice settings. The study is set within the City and County of Swansea and surrounding areas, an area comprising 8% (238 700 individuals) of Wales’ total population. Of this population, approximately 94% are White; 12% of Swansea’s local areas are categorized in the top 10% of deprived areas in Wales, according to the 2011 Census. In the year 2011, there were approximately 35 598 births in Wales, of which 7.7% (2725) occurred within Swansea. ^6^

The specific aims of the initial grant funders to the Growing Up in Wales study were to: examine neighbourhood conditions and their impact on obesity in pregnancy, investigate the use of routine data to predict pregnancy complications and infant health, investigate the impact of household and neighbour characteristics on unintentional childhood injuries and examine lifestyle factors in pregnancy and the health of the infant at age 12 months. Subsequent grants supported cohort follow-up and assessment of maternal and infant health service use.

## Who is in the cohort?

Growing Up in Wales is a prenatally recruited birth cohort study which commenced in November 2009. The initial aim was to recruit 1000 families by March 2015; however, the final cohort is composed of 819 families in total. Women were enrolled into the study during pregnancy and were later followed up when the infant approached age 1 year. If fathers were present at study visits, they too were able to be involved. We identified potential participants within the Abertawe Bro Morgannwg University (ABMU) Health Board through approaching women at antenatal clinics. Within the waiting areas, women were introduced to the study by a research assistant (RA) and provided with an information leaflet and study contact details. In addition, study information leaflets and posters were deposited throughout maternity clinics. Exclusion criteria included women under the age of 16 years and women residing outside Wales. Following initial contact, eligible participants were sent an in-depth information sheet which outlined the study aims, objectives and rationale, exclusion criteria, requirements of participation and the benefits of taking part. Approximately 3 days after posting the information sheet, RAs telephoned participants to answer any study-related questions and to arrange a baseline visit. Thereafter, the RA carried out a one-off baseline visit {mean participant gestation 27.4 weeks [standard deviation (SD) 8, range 5.6–38.6 weeks]} within the participants’ home and obtained informed written consent. During the visit, participants completed a questionnaire and detailed anthropometry and blood pressure (BP) readings (systolic and diastolic) were recorded. Participants were also provided with a 7-day diet diary, an accelerometer (to be worn for 7 consecutive days) and a blood glucose kit (with instructions to self-administer two fasting measures).

**Figure 1.**
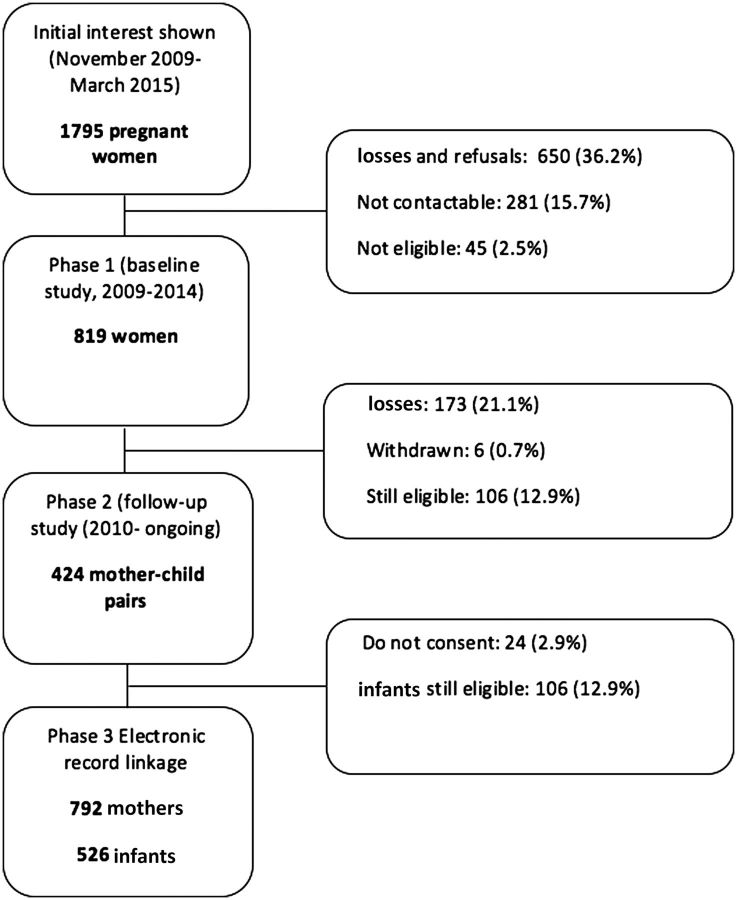
Flow chart of the Growing Up in Wales Birth cohort (2009–March 2015).

Figure 1 illustrates the flow of participants throughout the Growing Up in Wales study. Of the 1720 women approached by the RAs, 650 (36.2%) declined the option of participating, 45 (2.5%) did not meet the inclusion criteria and 281 (15.7%) could not be reached through further mail or telephone contact. Table 1 shows the distribution of deprivation categories of: (i) women who participated in the study; (ii) women who declined to take part; and (iii) women who showed initial interest but did not take part (e.g. not traceable or ineligible) (data available for 93.6% of women). As shown, approximately 35% of all women showing an interest in the study were in the least 50% deprived category. Similar distributions of deprivation were shown when comparing those women who declined to participate with those who enrolled in the study (42.5% vs 43.5% in 10–30% most deprived categories). Table 2 describes the characteristics of participants at the baseline visit.

**Table 1.** Socio-demographic characteristics of those women who participated, declined or were not contactable/eligible after initial recruitment

		Classification ^b^		
Deprivation group ^a^	Total study population	0	1	2
1	322	60	134	128
2	275	43	88	144
3	128	20	38	70
4	356	75	145	136
5	600	84	207	309
Missing				
Totals	1681	282	612	787

^a^ Welsh Index of Multiple Deprivation 2014 1 (10% most deprived)–5 (50% least deprived).

^b^ Classification 0 (not met inclusion criteria/not contactable), 1 (declined participation) and 2 (participated).

**Table 2.** Selected characteristics of study participants

Variable	*n*	Mean (SD) or %	Range
Maternal age at delivery (years)	783	29.03 (6.1)	16.6–44.7
Nulliparous	819	45%	0–6
Booking BMI (kg/m ^2^ )	557 ^a^	26.2 (6.3)	17–59.5
Fasting glucose (mmol/l)	183	6.03 (0.1)	3.4–8.2
Kessler 6 score ^b^	774		6–24
Ethnic group	783		
White/European		92%	
African/Caribbean		1.3%	
Asian		3.7%	
Other		2.9%	
Maternal education	787		
Higher		52.6%	
School		31.5%	
Trade		9.7%	
None		6.2%	
Working status	800		
Full-time		41.8%	
Part-time or casual		23.5%	
Unemployed		0.6%	
Homemaker		13.6%	
Other		20.5%	
Annual income	709		
£0 to £9 999		10.2%	
£10 000 to £14 999		10.7%	
£15 000 to £24, 999		13.8%	
£25 000 to £34 999		12.4%	
£35 000 to £39 999		8.2%	
£40 000 to £49 999		12.7%	
> £50 000		19.2%	
Not specified		12.8%	
Maternal smoking	772		
Yes		18.7%	
No		81.3%	
Alcohol consumption	778		
Yes		33.8%	
No		66.2%	

^a^ 452 (81.1%) clinically recorded and 105 (18.9%) self-reported.

^b^ Non-psychological distress score.

## How often have they been followed up?

RAs carried out a follow-up visit around the time of the infant’s first birthday (median age of 1 year 30 days). Visits mainly took place within the home setting, but participants were also provided with the option of attending our research clinic if more suitable. Mothers again provided written informed consent for anthropometric measures to be obtained from both herself and the infant. Paternal data was also collected if the father was present and consenting. Study protocols were reviewed and approved by the South East Wales Research Ethics Committee, and investigations were conducted according to the principles of the Declaration of Helsinki ^7^ and upheld the ethical principles of Swansea University.

In March 2015, a total of 424 (51.8%) follow-up visits had been conducted and 106 infants remained eligible for a future follow-up, having not yet reached age 1 year ( Figure 1 ). Mothers who participated in the 12-month follow-up visit were more likely to be school or university educated, in full-time employment and more affluent (see Table 3 below) compared with those who have not been followed up (those declining or not contactable).

**Table 3.** Characteristics of mothers who did and did not participate in the 12-month follow-up

Characteristic	No follow-up visit Mean(SD) or %	Follow-up visit Mean(SD) or %	*P* -value
*n*	179	424	
Maternal age (years)	30.4 (0.5)	29.3 (0.3)	n.s
Household salary < £35 000	60.1%	45.9%	**
Full time employment	37.9%	45.7%	**
Unemployed	18.4%	8.4%	**
Higher education or school-based qualifications	31.9%	50.5%	***
Nulliparous	45.1%	41.4%	n.s

n.s, not significant.

** *P* < 0.01, *** *P* < 0.01.

In most study analyses, we have used multiple imputations to account for missing data using chained equations. For a variable to be included in the imputation process, it was required to have a minimum of 60% complete data.

With participant consent, a large amount of follow-up data was gathered from postnatal clinical records and electronic health records. A study sticker on the participant’s antenatal notes signified to hospital clerical staff that these notes would be reviewed by the study team following the birth. A RA reviewed the participants’ newly filed postnatal records, gathering information on the delivery and newborn birth measures. Subsequent follow-up of mother-child pairs is conducted through access to electronic health records, with use of administrative and clinical records (described in further detail below).

## What has been measured?

Growing Up in Wales integrates both family and individual level data with use of routine electronic data linkage. Prospectively gathering antenatal and postnatal data has provided the opportunity to determine influences of the early-life exposures on later health outcomes. Table 4 provides a catalogue of variables included in the baseline and follow-up visit. Participants were able to provide consent for all or particular aspects of data collection. A detailed description of the baseline protocol has been published elsewhere. ^8^

**Table 4.** Summary of variables collected throughout the study period

Phase	Measurements
Baseline	**Questionnaire:** maternal age, ethnicity, parity, household income and benefits, education, smoking and alcohol consumption, birth plans **Antenatal data:** body mass index (at 12 weeks’ gestation), blood pressure readings, medications, family health history, ultrasound growth measures, previous pregnancies/deliveries, smoking and alcohol consumption during and before pregnancy, obstetric and family history **Anthropometric data:** parental height, weight, four skinfold thicknesses (biceps, triceps, subscapular and supra-iliac) and mid-arm circumference Blood pressure **Fasting blood glucose readings:** Self-administered, providing two readings over a 7-day period **Objective physical activity:** 7-day accelerometer (Actigraph worn around waist or wrist-worn GENEA accelerometer) **Dietary data:** 7-day diet diary **Housing assessment:** hygiene, presence and extent of mould and/or damp, household hazards, safety features, noise levels and temperature and humidity readings **Postnatal data:** duration of labour stages, birth type, birth place, infant birthweight, gestation, APGAR scores at 1 and 5 min, head circumference, length of hospital stay **Semi-structured interviews**
Birth	**Delivery data:** place of birth, delivery type, induction required, fetal presentation, length of labour stages, complications and pain relief **Infant data:** sex, date and time of birth, gestation, birthweight, head circumference, APGAR scores at 1 and 5 min, initial feeding method, feeding method on discharge and duration of hospital stay
Follow-up at infant’s first birthday	**Questionnaire:** Income, employment, maternity leave duration, home ownership, family dynamics, support networks, infant sleep and activity, developmental milestones, infant injuries, infant feeding history; breastfeeding duration, complementary feeding, age of weaning, infant and mother food frequency **Anthropometric data:** repeat all baseline measures for mother, infant length, weight, four skinfold thickness (biceps, triceps, supra-iliac and subscapular), head, mid-arm and abdominal circumferences **Dietary data:** 7-day diet diary for mother and infant **Objective physical activity:** 7-day accelerometer worn by mother and infant **Child health data:** routine growth measures (length, weight and date of assessment) and health assessment checks (vaccinations) **Semi-structured interviews**
Ongoing	All participants are followed using routine data linkage providing health service (general practice, inpatient, outpatient and prescription data), education and household data

A key exposure for many of the cohort analyses is maternal BMI during early pregnancy. This measure was obtained from antenatal records during the baseline visit, having been measured and recorded by a midwife during the routine booking visit (usually occurring at 12 weeks of gestation). Subsequent detailed anthropometric measures were obtained by the RA during the baseline and follow-up visit. Measures included height, weight, skinfold thicknesses (biceps, triceps, subscapular and supra-iliac) and head (infant only), mid-arm and abdominal circumferences. Each measure was obtained from the mother during pregnancy and from the mother and infant at the 12-month visit. Anthropometric measures at birth (weight, length and head circumference) and throughout the first year (weight and length measures) were also extracted from postnatal records and the child community health book (measures recorded by a health visitor).

Physical activity was objectively measured over a 7-day period during pregnancy and at the 12-month follow-up visit (for both mother and infant). Initial data were collected using a waist-worn Actigraph (GT3X) accelerometer ^9^ (baseline *n* = 141). The accelerometer collected step counts on a 1-epoch per s basis at a rate of 30 Hz, providing the total number of activity counts over the duration of 7 days. Data were used to estimate the participant’s average daily activity count. Containing a ‘wear time validation rule’, the software selected only days in which data displayed a minimum of 8 h of wear. Subsequently, to be included within analyses participants were required to have at least 1 valid day of data. With the later development of waterproof accelerometers and occasional reporting of discomfort with a belt-worn device, we discontinued using the Actigraph (GT3X) accelerometer in February 2013. Instead, participants (baseline *n* = 124, 12-month follow-up *n* = 116) were provided with a wrist-worn (non-dominant hand) waterproof GENEA accelerometer. ^10^ Previous findings ^11^ comparing the use of both accelerometers worn around the waist have shown almost identical accuracy between devices. Data from the accelerometer enabled us to classify the intensity at which a participant performed active tasks (defined as the ‘non sedentary SVM’). During the 12-month visit, the infant wore the GENEA accelerometer ^10^ around the left ankle ( *n* = 129).

Qualitative data collection provided contextual data to inform possible interventions, with specific foci on child injury prevention and family dietary choices. Semi-structured interviews ( *n* = 82) were carried out with purposive samples of prospective parents and parents of infants within the home setting. Interview schedules were guided by the socio-ecological model which suggests that health behaviours are predicted by the community and home environments and personal factors. Participants were selected to provide a representative sample of both affluent and deprived neighbourhoods and data were analysed using inductive thematic analysis.

Growing Up in Wales was designed to take advantage of the increasing availability of electronically-held, person-based, routinely collected data from consenting participants. In Wales, de-identified electronic records are stored at the Secure Anonymised Information Linkage (SAIL) databank which was previously developed within the Health Information Research Unit (HIRU) at Swansea University. ^12^^,^^13^ The SAIL databank enables record linkage of a wide range of pseudonymized, person-based data from health and other datasets and currently incorporates over 700 million rows of data concerning multiple health and social care events. A split-file approach to identity matching using multiple encryptions by different organizations replaces identifiers with unique numbers and facilitates embedding of cohorts and trials in a privacy-protecting remote analysis environment.13 The databank provides support for automatic longitudinal follow-up of mothers and infants in the Growing Up in Wales cohort. This study component aids cohort attrition and provides the opportunity to model study findings to the entire population. During both study visits, the RA explained: the data anonymization process; how routine data are accessed; that all data within the SAIL gateway are treated in accordance with the Data Protection Act 1998; ^14^ and that data access is managed by the Information Governance Review Panel (IGRP). Participants could then provide written informed consent if willing to participate in this component of the study. The main health datasets accessed for our cohort analyses include: the Primary Care Practice Clinical Systems (GP) database, the Patient Episode Database for Wales (PEDW); and the National Community Child Health Database (NCCHD). Table 5 provides an overview of data which can be accessed from each of these databases. Currently 792 mothers of 526 infants have provided consent for data linkage; however, the number of infants is set to increase with ongoing 12-month follow-ups.

**Table 5.** Datasets accessed within the SAIL database for Growing Up in Wales analyses

Database	Data type	Start date	Completeness	Inclusions
GP	Clinical	1993	100% of practices in Swansea	Data on patient signs and symptoms, investigations, test results, diagnoses, prescribed medications and referrals for specialist treatments
			76% of practices in Wales	
PEDW	Clinical and administrative	1991	100% (for all inpatient and day-case activity of individuals attending NHS hospitals in Wales)	Attendance and clinical records regarding all inpatient and outpatient activity (both elective and emergency records). Data on spell duration, episodes of care, diagnoses, operations, treatments and specialties accessed
NCCHD	Clinical and administrative	1987	100%	Data on both mother and child including place of birth, date and time of birth, gestational age, birthweight, child sex and age of mother at delivery.

GP, primary care practice clinical systems database; PEDW, Patient Episode Database for Wales; NCCHD, National Community Child Health Database; NHS, National Health Service.

## What has it found? Key findings and publications

In this section we summarize our key study findings to date, categorized by outcome.

### Childbirth outcomes

Physical activity level during pregnancy was associated with delivery type but not with offspring outcomes, e.g. birthweight, gestational age and APGAR scores. ^15^ We found that higher activity levels during pregnancy were associated with a reduced likelihood of women requiring an instrumental delivery, e.g. caesarean section, forceps or ventouse. In comparison, women with low activity levels were 72% more likely to require an instrumental delivery. These findings were independent of maternal BMI. Conversely, when examining the effects of maternal BMI on delivery and birth outcomes, no significant associations were evident with delivery type, despite overweight and obese women revealing a trend for increasing numbers of elective caesarean sections. Overweight and obesity in pregnancy was, however, associated with a greater risk for induction, longer hospital stay following childbirth and delivery of a large-for-gestational-age infant. These findings remained after controlling for maternal age, parity and smoking status during pregnancy.

Our findings have identified modifiable factors that are associated with independent adverse effects on delivery and infant outcomes. Requiring further support from randomized controlled trials, our findings suggest that interventions aiming to reduce the risk of instrumental births need to focus on increasing women’s physical activity levels before or during early pregnancy. Interventions which target positive outcomes for the newborn, i.e. healthy birthweight, should focus on excessive weight among women of child-bearing age.

### Maternal obesity and associated heath service costs

Extensive literature details the additional costs of providing care to obese individuals, with one particular paper reporting an approximate 2.3% increase in total direct healthcare costs for every unit increase in BMI in the general population. ^16^ In line with this general pattern, we found that caring for obese women utilizes an extra £1 200 of National Health Service (NHS) resources per pregnancy after adjusting for multiple confounding factors. ^17^ Ours appears to be the first study to examine health service usage and associated costs throughout pregnancy among a general population sample.

We found that increasing costs were due to an increased usage of health service provision across primary and secondary care sectors (general practitioner visits and prescriptions, inpatient visits and duration and outpatient visits), with obese women revealing a 15–20% greater usage of all hospital services. An evident limitation was our inability to consider how a woman’s BMI changed throughout the course of pregnancy. As our findings were reliant upon one recording of BMI (at 12 weeks of gestation), we cannot rule out the possibility of women changing BMI groups in later trimesters. However, our findings are likely to present a conservative view as we did not measure indirect and intangible health service costs.

Using the same sample of women, we have more recently examined the use of health service provision by infants from birth to age 1 year, to ascertain whether infants born to obese mothers require additional health services (publication awaiting journal). Examining the same health service outcomes as for mothers, our findings revealed that infants born to obese mothers had higher health service usage and associated costs. In conjunction with our earlier maternal cost estimates, overall cost analyses revealed that on average an obese woman and her child accrue an additional £2 310 of services during pregnancy and in the first year of life. With one in five women attending prenatal care being classed as obese in the UK, ^18^ and 778 805 births in the UK during 2013, ^19^^,^^20^ this equates to an estimated additional resource use of £360 million per annum. The implications of our findings are important for informing the design of cost-effective interventions and policies aimed at reducing the obesity during early-pregnancy.

### Factors influencing infant size

The Medical Research Council (MRC) guidelines for developing effective interventions state that an evidence base underpinning intervention design is required in order to plan individual and linked components of complex interventions. ^21^^,^^22^ Examining multiple factors of influence throughout the early life course and adopting a hypothesis-driven approach has enabled us to determine: (i) whether childhood obesity prevention methods should better target the prenatal, postnatal or both phases; and (ii) which components the intervention should target, in order to be most effective.

**Figure 2.**
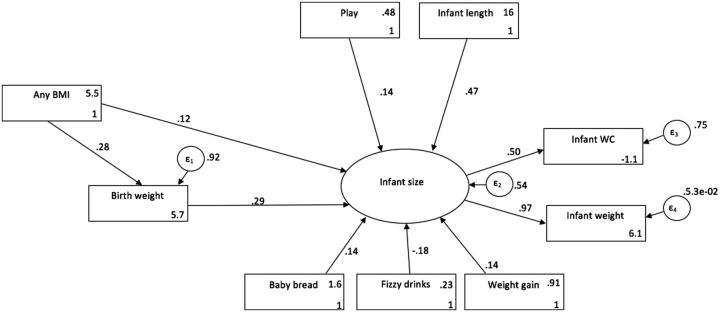
Modified structural equation model displaying standardised coefficients ( *X*_2_ (11) = 21.5, p < 0.05; RMSEA = .07; CFI = .94; SRMR = 0.05. Any BMI (mother's BMI at 12 weeks gestation), play (infant plays with parent daily or less often), Infant length (length at age 12 months), Baby bread (infant consumes carbohydrates daily or less often), Fizzy drinks (infant consumes carbonated drinks daily or less often), Weight gain (infant average weekly weight gain (g) from birth to 6 months), Infant size (factor of infant waist circumference (infant wc) and infant weight, both measure at age 12 months). Arrows indicate the pathway of associations with accompanying standardised coefficients. Residual error terms for endogenous variables are depicted by circles (ε _1_ –ε _4_ ). RMSEA, Root Mean Square Error of Approximation; CFI, Comparative Fit Index; SRMR, Standardized root mean square residual.

Figure 2 depicts our main study findings, ^23^ outlining results from a structural equation modelling approach. The figure includes all factors in our analyses and reveals important associations with infant size (latent variable of infant weight and waist circumference) at age 1 year. As shown, only one prenatal factor, maternal BMI, demonstrated a significant association with infant size, whereas several associations with postnatal factors are evident. Maternal BMI revealed both a direct and indirect effect on infant size, with an indirect effect moderated by birthweight. The consumption of carbonated drinks and lower levels of infant play were associated with smaller infant size. Conversely, greater consumption of carbohydrates, higher rate of weight gain and greater infant length predicted larger infant size. Unexpectedly, our model revealed that maternal factors such as age, ethnicity, education and income were not directly predictive of early infant size.

The results of this study are helpful in planning interventions, as all factors identified within our model are modifiable and open to testing in interventional designs. The components highlighted within our model suggest that future efforts aiming to prevent childhood obesity should perhaps focus on promoting healthy weight among women of child-bearing age, by encouraging healthy diets and active lifestyles, with continued support after childbirth.

### Parental perspectives: injury prevention in infancy and heathy eating

We found that mothers’ migration status was an influential factor in mothers’ knowledge of safety devices for child injury prevention, with newly migrated mothers not as aware of UK-based safety advice and available devices. ^24^ Lack of awareness surrounding a number of hazards such as the checking of bath water temperature, carrying hot drinks around children and using baby-walkers was quite common among the whole sample. When asked about facilitators and inhibitors of child safety devices and practices, mothers voiced the importance of social networks and British Safety Standard kitemarks as major facilitators. We also found that factors that inhibited uptake of child safety practices included child birth order, personality, stage of development, cost of devices and the adoption of alternative non-evidence-based strategies.

Future efforts to reduce child injury should provide specific, tailored messages to sub-groups of mothers; for example, affluent women reported the need for access to written materials whiereas mothers from deprived backgrounds preferred the use of posters and the media to disseminate safety messages. Our findings also emphasized the importance of delivering such messages in a timely manner appropriate to the stage of development of the child and the notion of having specifically trained individuals to work with families in the early years to raise awareness of childhood injury prevention.

Examining the main barriers of dietary choices faced by young families, ^25^ parents identified four predominant areas of influence: (i) provision of food choices, e.g. fast-food outlets and promotion of unhealthy food in supermarkets; (ii) implication of daily schedules, e.g. shift patterns and transport timetables; (iii) past experiences, e.g. own childhood dietary intake and education; and (iv) influence of family and friends, e.g. peer pressure. In the context of future interventions and policy-level decisions, our findings outline the need to develop population/community-level interventions aiming to reduce socio-ecological barriers to healthy dietary choices. Specific recommendations include: increasing access to affordable healthy food options within schools, workplaces and hospitals; educating parents on food preparation and storage; and reducing the prevalence of unhealthy fast-food outlets.

### What are the main strengths and weaknesses?

The primary strength of Growing Up in Wales is the combination of self-reported and objective measures supplemented by linkage to longitudinal maternal and child electronic health records. This particular study design provides a strong basis for undertaking life-course analyses on local data and complements other epidemiological cohort studies across the world, ^4^^,^^26–30^ allowing for collaboration through multi-cohort meta-analyses. A particular strength is the availability and accessibility of electronic data sets, reinforcing our ability to indirectly follow the health of participants and their offspring throughout the life span. Importantly, our findings are timely, portraying effects of the current obesogenic environment on health outcomes and rapidly feeding into policy making. Unlike much older cohorts collecting data during the war or post-war environment ^31–33^ or throughout the past decade, ^5^^,^^34^ our findings reflect relatively up-to-date exposures and may have greater relevance when developing interventions, given the technological advances and changing exposures, e.g. availability of convenience foods, throughout the 21st century. Embedding a cohort among a dynamic population-based multi-source data linkage environment will also inform and support the development and evaluation of a range of policies and interventions.

Weaknesses include the possibility of selection bias and the extent to which our findings are generalizable to the wider population, since all participants resided within the surrounding areas of Swansea City. However, this is common to all area-based cohorts. We must also acknowledge our relatively small sample size in comparison with larger cohort studies. ^34^ This ultimately reduces the statistical power to detect small but important findings and provides quite wide confidence intervals. The small sample size did however enable us to collect multiple in-depth variables and employ face-to-face collection methods. The future involves combing data from this cohort with many others in meta-analyses, to answer important generalizable questions.

## Can I get hold of the data? Where can I find out more?

We encourage collaborations and provide opportunities to access our electronic datasets remotely. All study data are stored within the Secure Anonymised Information Linkage (SAIL) databank at the Health Information Research Unit (HIRU) at Swansea University. All proposals to use SAIL datasets must comply with HIRU’s information governance policy. For more information please contact the principal investigator of the study, Professor Sinead Brophy, at [ S.Brophy@swansea.ac.uk ]. For further information please access our study website at [ www.EHL.swansea.ac.uk ].

Growing Up in Wales profile in a nutshell

- Growing Up in Wales is a prospective birth cohort study investigating the impact of gestational and postnatal environmental risk factors on infant health and development across the life course.
- A total of 819 pregnant women (aged 16–44 years), residing in and around the City of Swansea, UK, participated in a baseline visit during the period of November 2009–March 2015.
- Follow-up of mother-child pairs occurred as the infant reached age 12 months; 424 follow-ups have been carried out and 106 mother-child pairs still remain eligible.
- The dataset comprises questionnaires, anthropometrics, diet diaries, accelerometer data and linkage to individual-level (for both mother and infant) health, education and administrative records.
- Growing Up in Wales can provide data upon request and collaboration is welcome.

## Funding

This work was supported by Swansea University, Public Health Wales NHS Trust and the National Institute for Social Care and Health Research. The funders had no role in study design, data collection and analysis, decision to publish or preparation of the manuscript. The work was undertaken with the support of the Centre for the Development and Evaluation of Complex Interventions for Public Health Improvement (DECIPHer), a UKCRC Public Health Research Centre of Excellence. Joint funding (MR/KO232331/1) from the British Heart Foundation, Cancer Research UK, Economic and Social Research Council, Medical Research Council, the Welsh Government and the Wellcome Trust, under the auspices of the UK Clinical Research Collaboration, is gratefully acknowledged. This work was also supported by the Farr Institute. The Farr Institute is supported by a 10-funder consortium: Arthritis Research UK, the British Heart Foundation, Cancer Research UK, the Economic and Social Research Council, the Engineering and Physical Sciences Research Council, the Medical Research Council, the National Institute of Health Research, the National Institute for Social Care and Health Research (Welsh Assembly Government), the Chief Scientist Office (Scottish Government Health Directorates) and the Wellcome Trust, (MRC Grant No: MR/K006525/1).
